# Improving the readiness and clinical quality of antenatal care – findings from a quasi-experimental evaluation of a performance-based financing scheme in Burkina Faso

**DOI:** 10.1186/s12884-023-05573-x

**Published:** 2023-05-15

**Authors:** Inke Appel, Julia Lohmann, Manuela De Allegri, Jean-Louis Koulidiati, Serge Somda, Paul Jacob Robyn, Hermann Badolo, Stephan Brenner

**Affiliations:** 1 Heidelberg Institute of Global Health, Heidelberg University Hospital and Medical Faculty, University of Heidelberg, INF 130.3, 69120 Heidelberg, Germany; 2 Department of Gynecology and Obstetrics, Otto-Von-Guericke University, Magdeburg, Germany; 3 Department of Global Health and Development, London School of Hygiene and Tropical Medicine, London, UK; 4 Institut Supérieur Des Sciences de La Santé, Université Nazi Boni, Bobo-Dioulasso, Burkina Faso; 5 Centre Muraz, Institut National de Santé Publique (INSP), Bobo-Dioulasso, Burkina Faso; 6 Health, Nutrition and Population Global Practice, World Bank, D.C. Washington, USA

**Keywords:** Performance-based financing, Antenatal care, Quality of care, Burkina Faso

## Abstract

**Background:**

While maternal mortality has declined globally, it remains highest in low-income countries. High-quality antenatal care (ANC) can prevent or decrease pregnancy-related complications for mothers and newborns. The implementation of performance-based financing (PBF) schemes in Sub-Saharan Africa to improve primary healthcare provision commonly includes financial indicators linked to ANC service quality indicators. In this study, we examine changes in ANC provision produced by the introduction of a PBF scheme in rural Burkina Faso.

**Methods:**

This study followed a quasi-experimental design with two data collection points comparing effects on ANC service quality between primary health facilities across intervention and control districts based on difference-in-differences estimates. Performance scores were defined using data on structural and process quality of care reflecting key clinical aspects of ANC provision related to screening and prevention pertaining to first and follow-up ANC visits.

**Results:**

We found a statistically significant increase in performance scores by 10 percent-points in facilities’ readiness to provide ANC services. The clinical care provided to different ANC client groups scored generally low, especially with respect to preventive care measures, we failed to observe any substantial changes in the clinical provision of ANC care attributable to the PBF.

**Conclusion:**

The observed effect pattern reflects the incentive structure implemented by the scheme, with a stronger focus on structural elements compared with clinical aspects of care. This limited the scheme’s overall potential to improve ANC provision at the client level after the observed three-year implementation period. To improve both facility readiness and health worker performance, stronger incentives are needed to increase adherence to clinical standards and patient care outcomes.

**Supplementary Information:**

The online version contains supplementary material available at 10.1186/s12884-023-05573-x.

## Background

Although maternal mortality in Sub-Saharan Africa (SSA) decreased substantially since the early 2000s, in 2017 maternal health losses still remained high with 534 deaths per 100.000 live births [1]. Frequent antenatal care (ANC) visits improve maternal outcomes and reduce maternal and neonatal mortality [2, 3]. The basic components of ANC to ensure the wellbeing of mother and baby include pregnancy-specific health screenings and prevention of pregnancy-related risk factors, while more advanced aspects of ANC include the monitoring or management of those women at imminent risk of developing pregnancy-related complications [4]. High quality ANC therefore ensures timely management of hypertensive disorders, can prevent low birth weight in newborns, and increases the likelihood of women giving birth in a health facility, which is further linked to reduced perinatal mortality [5, 6].

ANC usually presents the first opportunity for women to access health services offered along the continuum of maternity care. Low ANC coverage, especially with respect to repeated ANC visits, as well as low quality clinical assessments are common factors limiting ANC’s potential to improve health outcomes for pregnant women. For instance, an assessment of ANC provision and service use in SSA between 2002–2014 found that while a majority of pregnant woman had access to basic ANC health screenings, still 18% and 35% of pregnant women did not undergo routine clinical blood or urine tests [7].

PBF schemes usually introduce additional health worker payments linked to defined performance measures to streamline the quality, effectiveness, and efficiency of ANC service provision by aligning health workers’ responsiveness and motivation with priority health service targets [8].A recent review of 59 PBF programs showed that PBF has the potential to improve the availability of ANC-relevant medicine and equipment as well as key care processes, such as the prescription of iron or folic acid to pregnant women, while a positive impact of PBF on the overall quality of ANC provision remains limited [9]. In Rwanda and Cameroon, higher proportions of women received anti-tetanus vaccinations during ANC visits after PBF implementation, while there was no overall effect on the content of ANC visits in the Cameroon PBF [10, 11]. In Burundi, PBF increased the chance of a pregnant woman’s blood pressure being measured [12]. In contrast, in the Republic of Congo PBF implementation resulted in a negative effect on the availability of vaccines and equipment [13].

Burkina Faso is a low-income country in SSA with a maternal mortality ratio in 2017 of 320 deaths/100,000 live births [14]. In 2011, the Government launched a pilot PBF scheme in three districts to improve primary health care provision at district and community levels to further reduce maternal and newborn mortality. In 2014, the Ministry of Health (MoH) with funding from the World Bank was able to geographically expand a revised design of the PBF scheme. The 2014 scheme was implemented in health facilities across six regions (Centre Nord, Centre Ouest, Nord, Sud Ouest, Boucle du Mouhoun, and Centre Est). In each region, the MoH identified two intervention districts based on their weaker performance with respect to key maternal health service outcomes. With respect to ANC, quantity indicators included the volume of services delivered to pregnant women attending ANC consultations and the number of pregnant women who received at least two tetanus vaccines during ANC. Quality indicators assessed the availability of key ANC equipment, supplies and drugs. With each payment cycle, facilities received defined fee-for-service payments for the volume of services provided for each quantity indicator and an additional financial bonus based on their achievement score computed across all quality indicators.

The objective of this study was to assess the extent to which this PBF affected the clinical quality of defined ANC components and to understand what clinical aspects of ANC service provision were most responsive to the PBF incentives implemented in Burkina Faso. To do so, we measured by both process and structural elements and defined three key dimensions of the quality of ANC.

## Methods

### Study rationale, design and sampling

To assess the effect of PBF on the quality of ANC this study followed a quasi-experimental controlled design with two data collection points (baseline and endline) to compare changes in ANC service provision observed at primary level PBF facilities between districts with PBF (intervention) and districts without PBF (control)..For this purpose, two additional districts comparable in terms of health indicators and health system structures like number of health care facilities to those of the PBF districts were identified in each of the six regions. In these control districts, a random sample of primary health care facilities was surveyed. At each facility, a minimum of five non-randomly selected ANC visits on the day of data collection were directly observed.

### Data collection

Data were collected at two time points: baseline (October 2013 – March 2014) and endline (April –June 2017). Trained data collection teams spent one day at each sampled facility to complete all survey questionnaires. For this study, we relied on data collected by two different questionnaires: (i) a facility assessment consisting of an inventory checklist collecting information on facilities’ infrastructural elements and health service inputs (including the number of ANC-trained staff, availability of ANC specific drugs or equipment, etc.); (ii) a direct ANC observation checklist completed for each observed ANC consultation collecting information on different clinical and non-clinical aspects of the provider–client interaction during a routine ANC visit, such as the assessment of the client`s current and obstetric history, physical examination, laboratory screening, or educational content. At each facility, a minimum of five non-randomly selected ANC visits on the day of data collection were directly observed. To control for non-observed variables that potentially affected our outcome estimates, we treated the repeated facility measurements as longitudinal data. However, as ANC clinics took place only on certain days in a week, information on ANC case observations was available for only 67% of facilities in the baseline sample (but 94% of facilities at endline). For this study, we therefore decided to include only those facilities for which both baseline and endline data on ANC case observations were available.

### Outcomes

Our conceptual approach to framing and defining the quality of ANC outcomes is based on Donabedian’s framework on elements of quality of care [15]. Our focus here is on clinical quality, i.e. inputs and processes related to effective ANC [16]. Inputs and processes considered as effective ANC were identified from the WHO’s Service and Readiness Assessment [17]; process elements were identified from a range of WHO recommendations and guidelines on ANC processes [4, 18, 19]. Given recommended process elements relating to routine screening and prevention differ between clients attending their first vs. a follow-up ANC visit [19]. This results in five composite measures each reflecting different aspects of ANC quality: service readiness, screening of first visit clients, screening of follow-up visit clients, prevention for first visit clients and prevention for follow-up visit clients.

The first composite indicator, “service readiness*”,* combines key input elements required for quality ANC provision at the facility level, such as the availability of qualified clinical staff, equipment, and supplies to deliver quality ANC services. This indicator consisted of 11 variables taken from the facility inventory checklist.

The following two composite indicators, “screening of first visit clients” and “screening of follow-up visit clients”, combine process elements measuring screening activities at the case level, such as focused client assessment, and physical and laboratory screening for pregnancy-specific risk factors or complications. These indicators consisted of 24 (first visit) and 15 (follow-up) variables taken from the direct observation checklist.

The next two composite indicators, “prevention for first visit clients” and “prevention for follow-up visit clients”, combine process elements measuring prevention activities at the case level such as medical prophylaxis, client information and education on healthy behaviors during pregnancy, and birth planning. These indicators consisted of 11 (first visit) and 11 (follow-up) variables taken from the direct observation checklist.

All variables consisted of binary data. Composites were formed by additive aggregation of equally weighted variable items within each indicator. To allow for easier comparability, we transformed the values of each outcome indicator to a range from 0 to 1 in relation to their observed value range. Details on the indicators included in each composite score are provided in Table 1 in the supplemental file.

**Table 1:** Key characteristics and their distribution across sampled cases by study arm and time point

	Baseline		Endline	
	PBF	Control	PBF	Control
Total number of cases observed:	1291	200	1442	268
ANC visit type	n (%)	n (%)	n (%)	n (%)
First Visit	435 (33.7%)	66 (33.0%)	386 (26.8%)	104 (33.9%)
Follow-up Visit	856 (66.3%)	134 (67.0%)	1056 (73.2%)	164 (66.1%)
Pearson Chi 2	0.037 (*p* = 0.85)		16.0 (*p* < 0.01)	
ANC provider skills	n (%)	n (%)	n (%)	n (%)
Qualified (3-year training)	253 (19.6%)	48 (24.0%)	444 (30.8%)	87 (32.5%)
Qualified (1-year training)	894 (69.3%)	134 (67.0%)	940 (65.2%)	143 (53.4%)
Not qualified	144 (11.1%)	18 (9.0%)	58 (4.0%)	38 (14.1%)
Pearson Chi 2	2.53 (*p *= 0.28)		47.6 (*p* < 0.01)	
Client parity	n (%)	n (%)	n (%)	n (%)
Not first pregnancy	286 (22.1%)	164 (82.0%)	1122 (77.8%)	201 (75.0%)
First pregnancy	1.005 (77.9%)	36 (18.0%)	320 (22.2%)	67 (25.0%)
Pearson Chi 2	1.76 (*p* = 0.18)		0.27 (*p* = 0.61)	
Client literacy	n (%)	n (%)	n (%)	n (%)
Illiterate	1103 (85.4%)	165 (82.5%)	1149 (79.7%)	218 (81.3%)
literate	188 (14.6%)	35 (17.5%)	293 (20.3%)	50 (18.7%)
Pearson Chi 2	1.17 (*p* = 0.28)		0.39 (*p* = 0.53)	
Client SES	n (%)	n (%)	n (%)	n (%)
Lowest 20%	270 (20.90%)	82 (41.0%)	352 (24.4%)	40 (14.9%)
Not lowest 20%	1021 (79.1%)	118 (59.0%)	1090 (75.6.%)	228 (85.1%)
Pearson Chi 2	38.7 (*p* = 0.00)		11.50 (*p* < 0.01)	
Client age	mean (SD)	mean (SD)	mean (SD)	mean (SD)
	25.04 (5.92)	25.8 (6.24)	25.3 (6.01)	25.6 (6.39)
t-test	0.82 (*p* = 0.45)		0.76 (*p* = 0.29)	
Consultation time (minutes)	mean (SD)	mean (SD)	mean (SD)	mean (SD)
	16.9 (9.70)	22.70 (10.60)	12.40 (7.08)	11.79 (6.12)
t-test	5.68 (*p* = 0.76)		-1.38 (*p* = 0.37)	

*ANC* Antenatal care, *PBF* Performance-based financing, *SES* Socioeconomic status, *SD* standard deviation

To model PBF effects on those composite indicators measured at the case-level, we identified a set of control variables we expected to independently affect the measured quality outcomes based on existing evidence in the literature. The control variables include provider`s training level, clients’ parity, literacy, and socio-economic status, client age, and consultation time [20–22].

### Analytical approach

We used descriptive statistics to compare sample sizes and characteristics over time. We used Pearson`s chi-squared test to identify differences in key characteristics for all sub-samples. To estimate the effect of PBF on the different ANC quality outcomes, we used a difference-in-differences approach based on linear regression. The assumption of parallel trends prior to intervention was confirmed using routine data from these study facilities for selected ANC indicators [23]. As treatment assignment occurred at district level, we clustered standard errors at that level. In light of the small number of district clusters, we applied wild bootstrapping to further adjusted standard errors [24]. Given the longitudinal nature of our data, we applied facility fixed effects to adjust the model for time-invariant facility characteristics. In estimating effect sizes for case-level outcomes, we further adjusted the models by the provider- and client-specific control variables outlined above. The resulting model specification is expressed by the following equation:

$$Y_{dfit}=\alpha_f+\beta{RBF}_{dfit}+\gamma t_t+\delta\left({RBF}_{dfit}\bullet t_t\right)+\phi X_{it}+\varepsilon_{dfit}$$

where *Y*_*dfit*_ is the value of the composite score of each outcome variable for case *i* at facility *f* in district *d* at time *t* = 0 for baseline and *t* = 1 for endline; *RBF*_*dfit*_ is a dummy variable which takes value 1 for a case *i* observed at a facility *f* empaneled under the RBF in district *d* at time point *t* and 0 otherwise; *t*_*t*_ is a dummy variable indexing the time points of data collection (0 = baseline, 1 = endline); *X*_*it*_ is the set of additional control variables in the case-level models; and *ε*_*dfit*_ is the error term. Coefficient α*f* represents facility fixed effects, coefficient *γ* the time fixed effect, and coefficient *δ* the DiD estimate for the resulting effect size attributable to the PBF intervention.

## Results

### Characteristics of sampled cases

The longitudinal sample included 351 primary level facilities, of which 297 were intervention and 54 were control facilities. Most of these facilities (88.2% in the PBF and 96.3% in the control arm) were in a rural setting (data not shown). Table 1 presents the distribution of ANC case characteristics for each treatment arm at baseline and endline. While the absolute numbers of observed cases increased slightly over time (due to adjustments in endline data collection approach to ensure more facilities were visited at their ANC clinic days), we observed a significant increase in observed follow-up cases in the PBF arm at endline compared with baseline. In the control group, the proportions of first to follow-up visits remained unchanged between timepoints. There was a significant difference in the distribution of cases by ANC provider qualification. While the proportions for each qualification type did not significantly differ at baseline (majority of cases attended by a qualified provider with 1-year training), we observed a higher proportion of cases attended by higher qualified providers (3-year training) at endline in the PBF arm. There was a higher proportion of clients belonging to the poorest quintile in the control arm at baseline compared with endline. Further, average ANC consultation time decreased over time in both study arms, but more so in the control arm.

### Distribution of composite scores and effect estimates

Table 2 presents the five outcome variables and their average composite scores by study arm and time point. As outlined above, the ANC service readiness score was computed at facility level, the ANC screening and prevention quality scores were computed at case level and further divided into first and follow up visit scores. While comparable across study arms at baseline, facilities on average only met about half of the standards measured readiness score. Facilities in the PBF arm gained on average about 0.1 point over time (0.53 to 0.64, *p* = 0.03) but remained almost unchanged in the control arm. The main drivers contributing to this score increase largely included items related to infection prevention and improved availabilities of supplies and drugs (data not shown).

**Table 2:** Outcome variables and their average score distributions by study arm and time point

	Baseline				Endline			
	PBF		Control		PBF		Control	
	mean	SD	mean	SD	mean	SD	mean	SD
Service Readiness ^a^	0.53	0.15	0.56	0.14	0.64	0.12	0.57	0.14
t-test			0.03	0.02				
Screening Quality^b^								
First Visit	0.53	0.16	0.56	0.14	0.64	0.12	0.57	0.14
t-test			-0.02	0.02				
Follow-up visit	0.61	0.14	0.57	0.10	0.61	0.15	0.65	0.14
t-test			-0.03	0.01				
Prevention Quality^b^								
First Visit	0.27	0.16	0.23	0.17	0.43	0.23	0.36	0.19
t-test			-0.03	0.02				
Follow-up visit	0.30	0.16	0.26	0.17	0.42	0.22	0.39	0.18
t-test			-0.04	0.02				

*ANC* Antenatal care, *PBF* Performance-based financing, *SD* Standard deviation

^a^score computed at facility level

^b^score computed at case level

Similarly, average scores on screening quality met on average about half of the criteria measured by these scores in both study arms, regardless of visit type. Significant increases in scores over time were only observed for follow-up cases in the control arm (i 0.57to 0.65, *p* = 0.01). Average prevention quality scores were on average lowest compared with the other composite measures and were on average with 0.3 points almost twice as high in cases observed at PBF facilities compared with controls at baseline. The time trend showed a strong paralleled increase of average preventions scores over time (on average by about 0.15 points for both first visit: 0.27 to 0.43, *p* = 0.02 and follow-up cases: 0.3 to 0.43, *p* = 0.02).

Table 3 shows the effect sizes estimated by our regression models for each composite score. Effect sizes were overall small. The largest effect size directly attributable to the PBF intervention was observed for the ANC readiness score with a statistically significant positive change of 0.1 score points or 10 percentage points ( 95% CI 0.04;0.18. All other changes were statistically not different from zero.

**Table 3:** Effect sizes and probabilities estimated by unadjusted and adjusted models for each outcome variable

	Adjusted DiD model *	
	Effect δ	(95%-CI)
Service Readiness ^a^	0.10	(0.04; 0.18)
Screening Quality^b^		
First Visit	-0.0	(-0.20; 0.15)
Follow-up visit	-0.08	(-0.18; 0.03)
Prevention Quality^b^		
First Visit	-0.01	(-0.22; 0.17)
Follow-up visit	-0.02	(-0.12; 0.09)

*ANC* Antenatal care, *CI* Confidence interval, *DiD* Difference-in-differences, *PBF* Performance-based financing

^*^Covariates (binary variables) used for model adjustment: provider qualification, length of the consultation time, clients’ literacy, age, parity, and socioeconomic status

^a^score computed at facility level

^b^score computed at case level

## Discussion

This study investigates the effects of a PBF scheme in Burkina Faso on the quality of ANC provision at rural health centers and clinics. This study contributes to the existing literature on PBF implementation in LMICs by presenting a comprehensive assessment of ANC quality outcomes. To avoid an analytical focus on single input and process indicators, which sometimes limit a more comprehensive approach to the assessment of healthcare quality, this study therefore approaches ANC quality along three broader outcomes (i.e., service readiness, screening and prevention),thus keeping the analytical focus on functional aspects of ANC provision [9].

### ANC Readiness Quality

While improvements in ANC readiness remained below standards of high quality (as measured by the score), this study found that the Burkina PBF produced statistically significant increase in the readiness score. Data further suggest that this effect was largely mediated by improvements in the readiness to prevent infections and to provide ANC-specific supplies and drugs. This finding is comparable to a similarly designed PBF implemented in Cameroon Tanzania, the Republic of Congo, and Malawi, where the main effects of the scheme occurred with respect to clinical equipment availability [11, 25–27].

The positive impact of the Burkina PBF on facilities’ readiness to provide primary healthcare was also observed for other primary care services, such as child health care [28]. It therefore seems that the Burkina PBF was successful in improving the availability of key inputs across incentivized services. Two PBF design features might have driven this effect on facility readiness: the selection of performance indicators with a strong focus on structural indicators related to facility and service readiness, as well as facilities’ contractual obligation to invest about 60% of their earned PBF revenue into activities that ensured continuous service operations [29].

### ANC Processes Quality

The findings of this study did not identify any substantial effects of the Burkina PBF on clinical ANC content measured by our screening and prevention scores. This lack of clinical care improvements is also reflected in the findings from similarly designed PBF schemes in Zimbabwe and Cameroon. In Zimbabwe, where baseline levels of ANC quality scores were similarly low as in this study setting, no effects of the PBF scheme on the overall ANC quality was found [30]. In Cameroon, ANC observed at control facilities even improved in overall content of care compared with PBF enrolled facilities, although facility readiness was positively affected by the PBF scheme [11]. Given the similarity of these PBF designs, it seems that these schemes would require additional incentive structures that specifically address the performance of clinical ANC aspects.

One such aspect as a potential target for future performance incentives might be client education during ANC as client education, scored much lower compared with the prevention component. A recent study conducted in ten low- and middle-income countries found that among the surveyed six routine components of ANC provision, “information on pregnancy complications” was provided least [31]. And a study from Rwanda found that only about 80% of ANC providers educated their clients on pregnancy-related danger signs, and only 50% provided essential information on newborn care [32]. The observed effect on service readiness in combination with absent effects on content of care in our findings further illustrate the disconnect between clinical inputs and processes. In the case of the Burkina PBF, this disconnect has also been found between service readiness and care processes related to incentivized outpatient care provided to children [33]. Given the weak correlation between the predictability of clinical performance based on the availability of service inputs across countries in SSA [34], the selection of performance-based incentives in PBF designs should probably have a stronger focus on clinical processes to produce more pronounced effects on overall service quality.

We noticed some features of the Burkina PBF design that might have limited the scheme’s potential to overcome this input-process disconnect. For instance, the relative weights attached to performance indicators related to ANC promoted a stronger focus on service readiness (e.g. a relative weight of 60 points for infection prevention availability) compared with the often more complex performance of key clinical content (e.g. This incentive bias towards input of care elements might therefore have limited providers’ attention to clinical performance [35]. Further, qualified health professionals in Burkina Faso tend to earn a comparatively high and constant income that usually covers all basic needs [36]. Hence, the additional individual income bonuses linked to performance might have been too low and to intransparent to act as sufficiently strong financial incentives.

In addition to design features, the scheme implementation in Burkina Faso also faced several challenges like delayed payments or not enough education about the complex PBF design thatmight have undermined its potential effects on clinical ANC performance [37, 38]. As a result, adoption of the scheme by health workers might have been too limited or variable to motivate performance efforts that would eventually translate into marked improvements in ANC quality.. The negative effect of payment delays on successful PBF implementations has also been reported from schemes in Benin and Tanzania [39, 40].

### Methodological limitations

This study has some limitations. First, as mentioned above, longitudinal data was available for only a sub-set of study facilities. To ensure that limiting the facility sample based on longitudinal data availability did not bias our overall analysis, a sensitivity analysis was conducted treating the data as repeated cross-sections including all facilities observed at each of the two timepoints as described in the supplemental file. For the results also see the supplemental file.

Another limitation to the analytical approach is the relatively low number of 24 district clusters, which results in estimating downward-biased standard errors threatening the robustness of themodeled differences-in-difference coefficients. To limit over-rejection of the null hypothesis of no PBF effect, wild bootstrapp was used on all standard errors to test the robustness of the estimates. Lastly, given that health programs usually mature over time until they reach full potential [41], the study period with three years might have been too short to observe any effects due to maturation. Therefore, the effects observed in this study only apply to this early implementation period, and effects might differ as the implementation proceeds.

## Conclusion

In assessing the impact of the PBF scheme in Burkina Faso on ANC quality, our findings indicate that significant effects resulted only with respect to ANC service readiness. After an implementation period of about three years, this scheme has not yet resulted in substantial improvements with respect to key clinical content of ANC provision. We therefore conclude that the design of incentive mechanisms with a stronger focus on service inputs as well as challenges faced during implementation might have limited the scheme’s potential to positively affect the clinical quality of ANC. With respect to PBF implementation, our findings therefore suggest that to improve health workers’ adherence to clinical standards of care, performance incentives and related performance weights might need to be more specifically designed to link clinical performance to desired clinical processes and related quality outcomes.

## Supplementary Information

**Additional file 1: Table S1. **Key characteristics and their distribution across sampled antenatal care cases by study arm and time point for all available data. **Table S2.** Outcomevariables and their average score distributions by study arm and time point for all available data. **Table S3.** Effect sizes and probabilities estimated by adjusted models for each outcome variable for all available data.

## Data Availability

The quantitative datasets analyzed in the current study is available upon request in the World Bank’s Central Microdata Catalogue. Baseline: https://microdata.worldbank.org/index.php/catalog/2761 Endline: https://microdata.worldbank.org/index.php/catalog/3504
