# Supplementary material for: Improving the readiness and clinical quality of antenatal care – findings from a quasi-experimental evaluation of a performance-based financing scheme in Burkina Faso
Source: BMC Pregnancy Childbirth. 2023 May 15;23:352. doi: 10.1186/s12884-023-05573-x (PMC10184361; doi:10.1186/s12884-023-05573-x)
Supplement: Supplementary file 1 — Additional file 1: Table S1. Key characteristics and their distribution across sampled antenatal care cases by study arm and time point for all available data. Table S2. Outcomevariables and their average score distributions by study arm and time point for all available data. Table S3. Effect sizes and probabilities estimated by adjusted models for each outcome variable for all available data. [file 12884_2023_5573_MOESM1_ESM.docx]

# Supplemental File

Overview and definition of outcome indicators

| ANC outcome indicator | Analytical level | ANC quality aspects considered |
| --- | --- | --- |
| Service readiness (11 items) | Facility | General readiness: general infrastructure (reliable water/electricity/communication/lab service), infection prevention (available sterilization device/ hand washing/ waste disposal)  ANC specific: ANC supplies (tests/drugs in stock), ANC staff/supplies (minimum staff, essential equipment) |
| Screening first visit cases (24 items) | Case | Focused history assessment: previous obstetric history (age, gestations, interruptions, previous premature births, stillbirth, previous hemorrhage, previous complicated deliveries), current obstetric history (provider asks about medication, LMP, bleeding, fever, headache/vision, edema, fatigue)  Focused physical assessment: vital parameters (provider checks weight, blood pressure, fetal heartbeat), physical parameters (provider checks conjunctives, edema, fetal size, presentation), diagnostic tests (RPR, HIV, Hb) |
| Screening follow-up cases (15 items) | Case | Focused history assessment: previous obstetric history (provider checks ANC card), current obstetric history (provider asks about medication, LMP, bleeding, fever, headache/vision, edema, fatigue)  Focused physical assessment: vital parameters (provider checks weight, blood pressure, fetal heartbeat), physical parameters (provider checks conjunctives, edema, fetal size, presentation) |
| Prevention first visit cases (11 items) | Case | Prevention: prescription/ information about folic acid/iron, information/ distribution of ITN)  Education: education pregnancy (diet, danger signs), education birth (education about emergency plan, SBA, birth, breastfeeding, family planning) |
| Prevention follow-up cases (11 items) | Case | Prevention: prescription/information about folic acid/iron, prescription of malaria treatment, provider explains importance of IPTp)  Education: education pregnancy (diet, danger signs), education birth (education about emergency plan, SBA, birth, breastfeeding, family planning) |

ANC = antenatal care, Hb= hemoglobin, ITN = insecticide-treated bed nets, IPTp = intermittent preventive treatment of malaria in pregnancy, LMP = last menstrual period, RPR = rapid plasma reagin, SBA= skilled birth attended

## Sensitivity analysis – Analytical approach

In our main analysis, we made the decision to include only facilities for which complete data on variables was available for each of the two time points. To understand the effect our decision might have had on the results, we conducted a sensitivity analysis using all available data as repeated cross-sections (instead of a longitudinal panel), including those facilities for which no data was available at baseline.

In this sample, 358 facilities were included at baseline, of which 304 facilities implemented the PBF program, and 54 were included in the control group. At endline 515 facilities were included, of which 398 implemented the PBF program and 117 served as controls. So, at endline, information from an additional 157 facilities was included. Our analytical model changed in so far as we now estimated the differences-of-difference between both groups over time without controlling for unobserved fixed effects at the facility level.

Distribution of sampled cases

Table S1 presents the distribution of sampled antenatal case characteristics for each treatment arm at baseline and endline. The total number of observed cases in the PBF arm at baseline was 1,320 and in the control group it was 200. At endline, there were 1,920 cases in the PBF arm and 578 in the control arm. Obviously, the number of observed cases was substantially higher in the PBF arm and overall increased at endline for both study arms reflecting the increase in sampled facilities that offered ANC at endline.

A comparison of the frequencies at endline for each characteristic shows no major differences between the two analyses.

Table S1: Key characteristics and their distribution across sampled antenatal care cases by study arm and time point

|  | Baseline | | Endline | |  |
| --- | --- | --- | --- | --- | --- |
|  | PBF | Control | PBF | Control |  |
| Total number of cases observed: | 1320 | 200 | 1920 | 578 |  |
| ANC visit type | n (%) | n (%) | n (%) | n (%) |  |
| First Visit | 447 (33.9%) | 66 (33.0%) | 526 (27.4%) | 196 (33.9%) |  |
| Follow-up Visit | 873 (66.1%) | 134 (67.0%) | 1394(72.6%) | 382 (66.1%) |  |
| Pearson Chi 2 | 0.058 (p=0.81) | | 9.17 (p=0.002) | |  |
| ANC provider | n (%) | n (%) | n (%) | n (%) |  |
| Qualified (3-year training) | 253 (19.2%) | 48 (24.0%) | 633(33.0%) | 139(24.1%) |  |
| Qualified (1-year training) | 918(69.6%) | 134 (67.0%) | 1190(62.0%) | 367(63.5%) |  |
| Not qualified | 149(11.3%) | 18 (9.0%) | 97 (5.0%) | 72(12.4%) |  |
| Pearson Chi 2 | 3.04 (p=0.22) | | 47.6 (p<0.01) | |  |
| Patient parity | n (%) | n (%) | n (%) | n (%) |  |
| Not first pregnancy | 1.029(78.0%) | 164 (82.0%) | 1501(78.2%) | 446(77.2%) |  |
| First pregnancy | 291 (22.0%) | 36 (18.0%) | 419(21.8%) | 132(22.8%) |  |
| Pearson Chi 2 | 1.68 (p=0.19) | | 0.27 (p=0.61) | |  |
| Patient literacy | n (%) | n (%) | n (%) | n (%) |  |
| Illiterate | 1126(85.3%) | 165 (82.5%) | 1555 (81.0%) | 484 (83.7%) |  |
| literate | 194 (14.7%) | 35 (17.5%) | 365(19.0%) | 94(19.3%) |  |
| Pearson Chi 2 | 1.07 (p=0.30) | | 2.24 (p=0.14) | |  |
| Patient SES | n (%) | n (%) | n (%) | n (%) |  |
| Lowest 20% | 274(20.8%) | 82 (41.0%) | 481(25.1%) | 83(14.4%) |  |
| Not lowest 20% | 1046(79.2%) | 118 (59.0%) | 1439(77.4%) | 495 (85.6%) |  |
| Pearson Chi 2 | 39.68 (p<0.01) | | 29.06 (p<0.01) | |  |
| Patient age | mean (SD) | mean (SD) | mean (SD) | mean (SD) |  |
|  | 25.04 (5.93) | 25.8 (6.24) | 25.4 (6.10) | 26.20 (6.40) |  |
| t-test | 0.82 (p=0.45) | | 0.76 (p=0.29) | |  |
| Consultation time (minutes) | mean (SD) | mean (SD) | mean (SD) | mean (SD) |  |
|  | 17.05 (9.94) | 22.70 (10.60) | 12.50 (6.90) | 11,13 (5.65) |  |
| t-test | 5.68 (p=0.76) | | -1.38 (p=0.37) | |  |

Composite scores

Table S2 presents the three outcome variables and their average score distributions by study arm and time point for the expanded sample.

While readiness in the PBF arm increased by about 0.1 points over time (0.54 to 0.64), the score for the control group remained almost unchanged.

Scores for Screening Quality in follow-up cases were on average higher compared with first visit cases, with a statistically significant difference between cases observed in the PBF compared (0.61) with the control arm (0.57). Obvious changes over time were only observed for follow-up cases in the control arm (increase by 0.7 points up to 0.64).

Across cases, Prevention Quality scored on average lowest compared with the other scores, but also showed the strongest increases of all scores over time (on average by about 0.2 points for both first visit and follow-up cases. For first visit cases we found a statistically significant difference between average scores between PBF and control cases at baseline.

The analysis with the complete sample shows for the ANC Screening Quality Score in first visits cases a 0.14 points lower score distribution at endline in the PBF group compared with the main analysis. The other scores do not differ substantially between the two analyses.

Table S2: Outcome variables and their average score distributions by study arm and time point.

|  | Baseline | | | | Endline | | | |
| --- | --- | --- | --- | --- | --- | --- | --- | --- |
|  | PBF | | Control | | PBF | | Control | |
|  | mean | SD | mean | SD | mean | SD | mean | SD |
| ANC Service Readiness Score ^a^  t-test | 0.54 | 0.15 | 0.5  0.26 | 0.14  0.21 | 0.64 | 0.12 | 0.55 | 0.14 |
| ANC Screening Quality Scores ^b^ |  |  |  |  |  |  |  |  |
| First Visit  t-test | 0.53 | 0.16 | 0.51  -0.02 | 0.14  0.19 | 0.50 | 0.17 | 0.52 | 0.14 |
| Follow-up visit  t-test | 0.61 | 0.14 | 0.57  -0.03 | 0.10  0.01 | 0.61 | 0.15 | 0.64 | 0.15 |
| ANC Prevention Quality Scores ^b^ |  |  |  |  |  |  |  |  |
| First Visit  t-test | 0.27 | 0.16 | 0.23  -0.04 | 0.17  0.02 | 0.45 | 0.23 | 0.39 | 0.22 |
| Follow-up visit  t-test | 0.30 | 0.16 | 0.26  -0.04 | 0.17  0.15 | 0.42 | 0.22 | 0.40 | 0.18 |

ANC = antenatal care; PBF = performance-based financing; SD = standard deviation.

^a^ score computed at facility level; ^b^ score computed at case level.

Effect Estimates

Table S3 shows the effect sizes estimated by our DiD models for each measured score. Effects were overall weak. The largest effect size was observed for the ANC Readiness Score, with a statistically significant positive change of 0.08 points directly attributable to the PBF intervention.

The analysis of the complete sample shows a positive effect size of 0.05 points for the ANC Prevention Quality Score in first visits. In the main analysis the effect size for this category is negative (-0.01 points). Apart from that there are no major differences between the two analyses.

Table S3: Effect sizes and probabilities estimated by adjusted models for each outcome variable.

|  | Adjusted DiD model * | |
| --- | --- | --- |
|  | Endline | |
|  | Effect δ (95%-CI) | |
| ANC Service Readiness Score ^a^ | 0.08 (0.03; 0.13) | |
| ANC Screening Quality Scores ^b^ |  |  |
| First Visit | -0.01 | (-0.09; 0.06) |
| Follow-up visit | -0.03 | (-0.09; 0.03) |
| ANC Prevention Quality Scores ^b^ |  |  |
| First Visit | 0.05 | (-0.04; 0.14) |
| Follow-up visit | 0.01 | (-0.07; 0.09) |

ANC = antenatal care; CI = confidence interval; DiD = difference-in-differences; PBF = performance-based financing.

* Covariates (binary variables) used for model adjustment: provider qualification, length of the consultation time, clients’ literacy, age, parity, and socioeconomic status

^a^ score computed at facility level;

^b^ score computed at case level
